# GRP78 modulates cell adhesion markers in prostate Cancer and multiple myeloma cell lines

**DOI:** 10.1186/s12885-018-5178-8

**Published:** 2018-12-18

**Authors:** Christopher N. Cultrara, Stephen D. Kozuch, Poornema Ramasundaram, Claudia J. Heller, Sunil Shah, Adah E. Beck, David Sabatino, Jenny Zilberberg

**Affiliations:** 1 Department of Chemistry and Biochemistry, 400 South Orange Avenue, South Orange, NJ 07079 USA; 2 Center for Discovery and Innovation, Hackensack University Medical Center, 340 Kingsland Street, Building 102, Nutley, NJ 07110 USA

**Keywords:** GRP78, Gene knockdown, Epithelia-mesenchymal transition (EMT), Cell adhesion

## Abstract

**Background:**

Glucose regulated protein 78 (GRP78) is a resident chaperone of the endoplasmic reticulum and a master regulator of the unfolded protein response under physiological and pathological cell stress conditions. GRP78 is overexpressed in many cancers, regulating a variety of signaling pathways associated with tumor initiation, proliferation, adhesion and invasion which contributes to metastatic spread. GRP78 can also regulate cell survival and apoptotic pathways to alter responsiveness to anticancer drugs. Tumors that reside in or metastasize to the bone and bone marrow (BM) space can develop pro-survival signals through their direct adhesive interactions with stromal elements of this niche thereby resisting the cytotoxic effects of drug treatment. In this study, we report a direct correlation between GRP78 and the adhesion molecule N-cadherin (N-cad), known to play a critical role in the adhesive interactions of multiple myeloma and metastatic prostate cancer with the bone microenvironment.

**Methods:**

N-cad expression levels (transcription and protein) were evaluated upon siRNA mediated silencing of GRP78 in the MM.1S multiple myeloma and the PC3 metastatic prostate cancer cell lines. Furthermore, we evaluated the effects of GRP78 knockdown (KD) on epithelial-mesenchymal (EMT) transition markers, morphological changes and adhesion of PC3 cells.

**Results:**

GRP78 KD led to concomitant downregulation of N-cad in both tumors types. In PC3 cells, GRP78 KD significantly decreased E-cadherin (E-cad) expression likely associated with the induction in TGF-β1 expression. Furthermore, GRP78 KD also triggered drastic changes in PC3 cells morphology and decreased their adhesion to osteoblasts (OSB) dependent, in part, to the reduced N-cad expression.

**Conclusion:**

This work implicates GRP78 as a modulator of cell adhesion markers in MM and PCa. Our results may have clinical implications underscoring GRP78 as a potential therapeutic target to reduce the adhesive nature of metastatic tumors to the bone niche.

**Electronic supplementary material:**

The online version of this article (10.1186/s12885-018-5178-8) contains supplementary material, which is available to authorized users.

## Background

Tumor cells that reside or metastasize to the bone and BM can develop pro-survival interactions with stromal cells, including osteoblasts (OSB) at the endosteum, through adhesion molecules. Evidence suggests that recurrent and resistant malignant stem cells can remain relatively protected within the bone microenvironment during treatment and later re-initiate growth [1–3]. In particular, we have shown that N-cad is a necessary mediator of CD138^+^ patient-derived multiple myeloma (MM) cells adhesion to the endosteum, and that down-regulation of N-cad in OSB decreased MM-OSB adhesive interactions, restricting the ex vivo survival of these tumor cells [4]. These adhesive interactions are considered to be major factors by which cancer cells remain “dormant” and escape the cytotoxic effects of therapeutic agents. The mechanism of drug resistance has been described in MM, as well as in disseminated/metastatic prostate cancer (PCa) cancer [5–7]. Bone is a preferred site for PCa metastases, and currently no curative treatments exist once the tumor is established within this niche [8–10].

The 78 kDa glucose-regulated protein (GRP78) is a chaperone protein that serves as the main sensor for misfolded proteins in the endoplasmic reticulum (ER) and triggers the unfolded protein response (UPR) [11]. Additionally, GRP78 regulates intracellular signaling events associated with embryonic development, aging, Ca^2+^ homeostasis and insulin/IGF-1 signal transduction [11]. GRP78 is expressed ubiquitously in all cell types, and is located primarily in the endoplasmic reticulum where it chaperones protein folding activity, in the mitochondria where it interacts with pro-survival and apoptotic executors and at the cell surface where it directs cell signaling [12]. In cancer, GRP78 overexpression leads to a variety of signaling pathways associated with tumor initiation, proliferation, adhesion and invasion [12, 13]. Importantly, GRP78: 1) is highly active in osteoblastic, androgen-independent prostate cancer [14], suggesting that it might play a pivotal role in the interaction of PCa cells with OSB, 2) plays a critical role in the adhesion and invasion of hepatocellular carcinoma [15] and MM [16], 3) can mediate resistance against cytotoxic chemotherapy in PCa cells [17], and 4) is overexpressed in a quiescent MM cell sub-population resistant to treatment [18, 19]. Of note, GRP78 has been correlated with the expression of N-cad, E-cad and β-catenin, respectively in colon cancer and hepatocellular carcinoma [15, 20, 21]. While high levels of N-cad have been linked to poor prognosis of MM patients [22, 23] and to PCa metastasis and castration resistance [24], no studies have examined the potential interplay between GRP78 and N-cad in MM and PCa for modulating tumor-bone adhesion.

GRP78 overexpression can affect the EMT signaling pathways related to Snail/Slug and TGF-β/smad. These pathways are closely associated with metastasis of epithelial tumors [15, 21]. Classically, an EMT is associated with the upregulation of mesenchymal markers such as N-cad and vimentin along with parallel downregulation of the epithelial marker E-cad [25, 26]. In this study, we described a novel correlation between GRP78 and N-cad in MM and PCa cells where GRP78 KD induced the concomitant downregulation of both N-cad and E-cad, which decreased the adhesive interactions of metastatic PC3 cells with OSB. These results may contribute to a better understanding of the underlying survival properties conferred by cell-cell adhesions, aiding in the development of more effective therapeutic strategies against cancers that interact with the bone niche [9, 27–30].

## Methods

### ONCOMINE data mining

The ONCOMINE repository (https://www.oncomine.org/) is a repository of cDNA microarrays [31]. We searched ONCOMINE using the following filters: Gene: HSPA5 (GRP78) or CDH2 (N-cad), Analysis Type: Cancer vs. Normal Analysis, Cancer Type: prostate cancer or multiple myeloma. A summary table containing fold change and significance (*P* < 0.05) for each comparison, are presented as log2 median-centered intensity as reported by ONCOMINE. References from independent studies presented can be found in the Additional file 1.

### Cell culture

MM.1S (MM cell line, ATCC®CRL-2974), MM.1R (MM cell line, ATCC® CRL-2975), RPMI 8226 (ATCC® CCL-155), PC3 (bone metastatic PCa cell line, ATCC®-CRL-1435), and hFOB 1.19 (OSB cell line, ATCC® CRL-11372) were purchased from ATCC. MM cell lines were cultured in high glucose RPMI-1640 medium supplemented with 15% fetal bovine serum (FBS), 2.5 mM of L-glutamine and 1% penicillin/streptomycin. PC3 cells were cultured in RPMI-1640 medium containing 10% FBS, 2.5 mM of L-glutamine and 1% penicillin/streptomycin. Both cell lines were cultured at 37 °C in a humidified tissue culture incubator containing 5% CO_2_. hFOB 1.19 cell medium consisted of 1:1 mixture of Ham’s F12 Medium Dulbecco’s Modified Eagle’s Medium, with 2.5 mM L-glutamine, 10% FBS and 0.3 mg/ml G418 (Sigma-Aldrich). These cells were maintained and propagated at 33 °C and 5% CO_2_ except during co-culture experiments which were conducted at 37 °C. All cell lines were periodically checked for mycoplasma using MycoAlert™ Mycoplasma Detection Kit (Lonza). Authentication of cell lines was performed by STR DNA profiling analysis conducted by the Molecular Resources Facility at Rutgers University. Cell populations were frozen after 3 passages from the time of initial receipt and growth and were discarded after 30 passages.

### ER stress induction

For ER stress induction, MM.1S cells were seeded at a density of 7.5 × 10^5^–1.0 × 10^6^ cells/well in a 24-well plate. Cells were treated for 18 h with 10 nM bortezomib (BTZ) (Caymen Chemicals), 1 nM thapsigargin (Tg) (Sigma-Aldrich) or dimethyl sulfoxide (DMSO, Sigmal-Aldrich) as vehicle controls. Total RNA was isolated and subsequently analyzed via qRT-PCR.

### siRNA transfection

HSPA5 (GRP78) targeting siRNAs were purchased from Ambion (Carlsbad, USA). Two different siRNAs; *Silencer®* Select Pre-designed siRNA s6979 (5’ UUC UGG ACG GGC UUC AUA Gtt 3′) and s6980 (5’ UCU AGU AUC AAU GCG CUC Ctt 3′) targeting exons 6 and 8, respectively, were tested. For control, the *Silencer®* select negative control No. 2 siRNA was used (Ambion). siRNA transfections were performed using a modified reverse transfection technique [32] using a cocktail containing equimolar quantities of each GRP78 siRNA to maximize silencing potential. The GRP78 siRNA cocktail (or siRNA control) was diluted in Opti-MEM reduced serum medium and incubated with the TransIT-X2 dynamic delivery system (Mirus Bio) according to the manufacturer’s protocol. The siRNA-TransIT-X2 complexes were added to wells of either a 6- or 24- well plate upon which either MM or PC3 cells seeded in complete growth medium at a cell density of 7.5-9 × 10^5^ cells/well (6 well plate) or 0.75-1 × 10^5^ cells/well (24 well plate). GRP78 siRNA cocktail or control siRNA were used at a final concentration of 50 nM for PC3 and 100 nM for MM cell lines.

### RNA isolation and qRT-PCR

Total RNA was isolated following transfections (48 h) from TriZol (Ambion) preserved cells using a TriRNA Pure Kit (Geneaid), following the manufacture’s instructions. The collected RNA was quantitated on a Qubit 3.0 fluorimeter using the Qubit Broad Range (BR) assay kit (Thermo Fisher Scientific). RNA (200 ng) was reverse transcribed into cDNA using a high capacity cDNA kit (Applied Biosystems). RT-PCR was performed using pre-developed TaqMan™ gene expression primer-probes for GRP78 (assay ID Hs99999174_m1), N-cad (assay ID Hs00983056_m1), GRP94 (assay ID Hs00437665_g1), GRP75 (Hs00269818_m1), and GAPDH (Hs99999905_m1) and TaqMan™ fast advanced master mix. qPCR fast assay was carried out on a StepOnePlus RT-PCR system (Applied Biosystems). Fold changes were calculated with the ΔΔCt method using GAPDH as endogenous control and the negative siRNA as the control sample.

### Western blot

Total protein was isolated from the cell cultures following transfection (78 h). Protein lysates were prepared by lysing the cells in ice-cold RIPA buffer (G-Biosciences) supplemented with protease and phosphatase inhibitors (Millipore Sigma) which were diluted 1:10 as per the manufacturer’s recommendations. Cell debris was removed by centrifugation at 16,000×g at 4 °C and protein concentrations were determined using a Pierce™ BCA kit (Thermo Fisher Scientific). A sample (20–35 μg) of the supernatant protein was mixed with LDS buffer and DTT, incubated at 70 °C for 10 min and resolved on a 4–12% Bis-Tris PAGE gradient gel before being transferred to a PVDF membrane. Following transfer, the membrane was blocked in 5% skim milk for 1 h, washed and incubated at 4 °C overnight with a rabbit 1° mAb against human GRP78, GRP94, GRP75, N-cad, E-cad, TGF-β1, Slug, B-catenin or GAPDH (all purchased from Cell Signaling Technology) at a 1:1000 dilution. The membrane was subsequently washed and incubated with an anti-rabbit HRP-conjugated 2° Ab (Cell Signaling Technology) for 1 h at room temperature at 1:2000 dilution. The bands were visualized using a SignalFire™ ECL reagent (Cell signaling Technology) on a ProteinSimple FluorChem E imager. No changes in GAPDH band intensity between control siRNA and GRP78 siRNA were detected, therefore target protein bands were normalized against the loading control GAPDH.

### Flow cytometry and cell viability

Cell viability was assessed using an Annexin V/PI kit (Biolegend). Annexin V and PI were added to the cell samples post-transfection at 24, 48, and 72 h according the manufacturer’s recommendation, incubated for 15 min at room temperature in the dark, and followed by immediate analysis by flow cytometry (FC500 flow cytometer, Beckman Coulter). Data was processed with Kaluza® (Beckman Coulter) flow analysis software.

### Cell morphology assay

For cell morphology analysis, PC3 cells were transfected with GRP78 siRNA and harvested 48 h later or treated overnight with 20μg/mL of a N-cad neutralizing monoclonal antibody, clone CG-4 (N-cad NAb, Sigma-Aldrich) prior to analysis. The cells were counted on Z2 Particle Counter and Size analyzer (Beckman Coulter) and re-seeded at 20,000 cells/well in a standard 96 well plate and left to culture for an additional 18 h. The wells were subsequently washed and imaged under bright field on a Cell Insight CX5 high content screening instrument (Thermo Fisher Scientific). Images were analyzed using ImageJ software package (NIH). 15 fields of view (5 each from 3 independent experiments) of control cells or cells treated with GRP78 siRNA were analyzed using the ImageJ software package (NIH). Cells with a clearly defined spherical and darker border (under bright field) were considered as rounded. Morphology was calculated as rounded: N_rounded cells_/N_total_ cells or elongated: [N_total cells_-N_rounded cells_]/N_total cells_ and represented as a mean percentage ± SD.

### Adhesion assay

To determine the effects of GRP78 KD on PC3 cells adhesion to bone, PC3 cells were transfected with GRP78 siRNA and harvested 48 h later and cultured with hFOB 1.19 cells plated in a 96 -well plate until confluent. To track the PC3 cells in co-culture, transfected cells were labeled with the Vybrant CFDA SE (green) Cell Tracer Kit (5 μM) (Thermo Fisher Scientific) counted on a Z2 particle counter and size analyzer (Beckman Coulter), and then seeded at 10,000 cells/well onto confluent OSB. For comparison, parallel co-cultures were treated overnight with 20μg/mL of a N-cad NAb, clone CG-4 (Sigma-Aldrich). Number of fluorescently labeled cells [N_coculture_] were counted by high content screening (Cell Insight CX5, Thermo Fisher Scientific) following an 18 h co-incubation period. Supernatants were then transferred from co-culture wells into empty ones to determine the number of fluorescently labeled floating cells [N_supernatant_]. Adherence was calculated as %N_supernatant_/ N_co-culture_.

### Statistical analysis

All data was plotted and analyzed using the GraphPad Prism software, V 7.0d (La Jolla, CA). Each experiment was performed in triplicates (*N* = 3). Data is represented as the mean ± SD. Comparisons between two groups were analyzed using unpaired student’s *t*-tests. A probability (P) value of less than 0.05 was considered statistically significant.

## Results

### GRP78 silencing leads to concomitant N-cad downregulation in MM.1S and PC3 cells

**Fig. 1:**
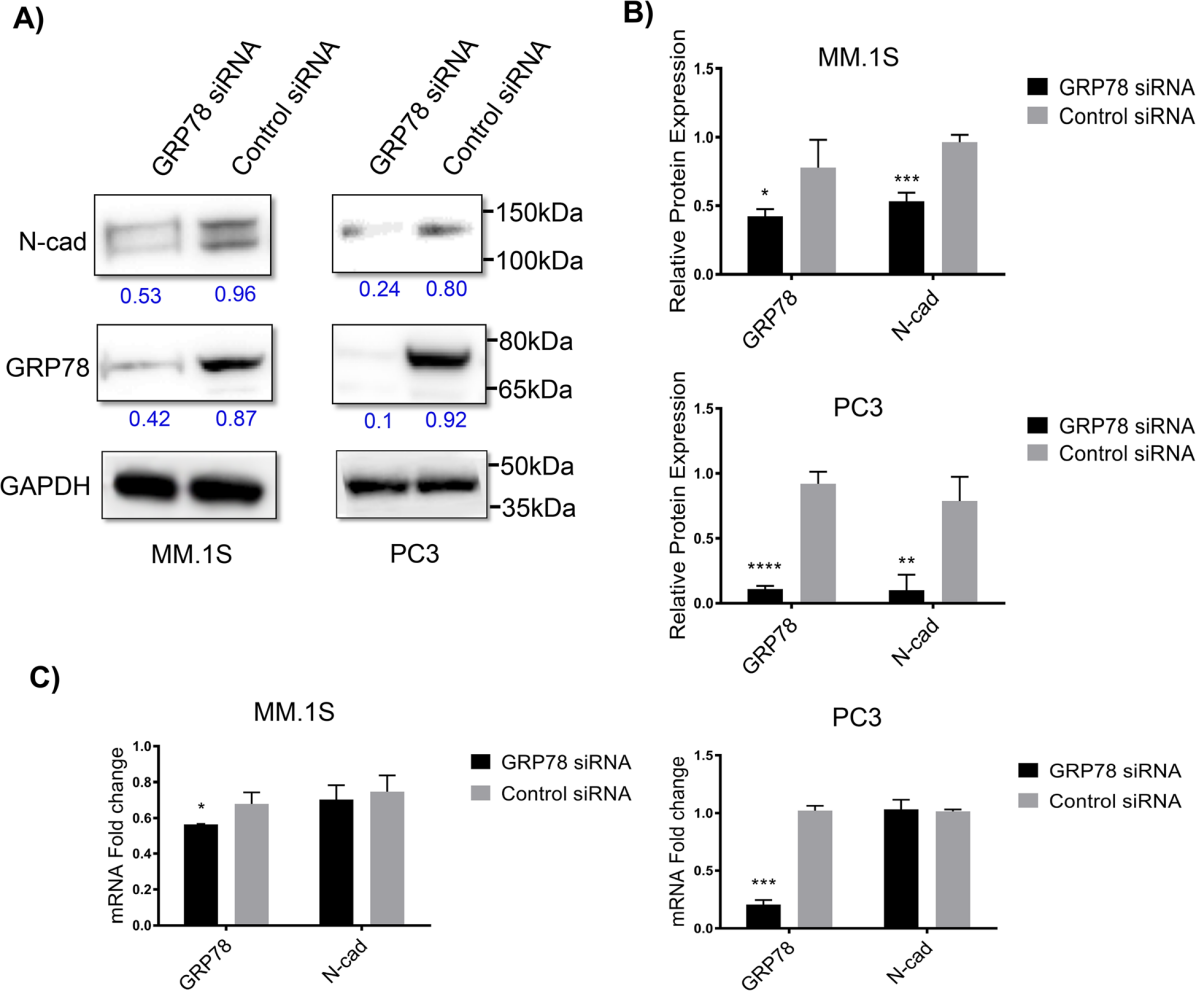
Relationship between GRP78 and N-cad in MM.1S and PC3 cells upon GRP78 KD. 100 nM and 50 nM siRNA cocktail against GRP78 or a control siRNA were transfected into MM.1S and PC3 cells, respectively. Total mRNA and protein levels were analyzed at 48 h and 72 h, respectively. **a** Western blots of endogenous GRP78 and N-cad protein expression after GRP78 silencing. **b** Protein expression quantification. Target protein levels were normalized against the loading control GAPDH and compared with the control, non-targeting siRNA. Blot bands and quantitative values are presented as the mean ± SD and representative of 3 separate trials. Western blot analysis for MM.1S and PC3 cells were performed independently. *P < 0.05 and ***P < 0.001 in MM.1S cells and ****P < 0.0001 and **P < 0.01 in PC3 cells for GRP78 and N-cad, respectively. **c** qRT-PCR analysis of relative mRNA levels for GRP78 and N-cad upon GRP78 silencing. Target mRNA levels are relative to the control siRNA and represented as the mean fold change ± SD of 3 separate trials. *P < 0.05 in MM.1S cells and ***P < 0.001in PC3 cells for GRP78

To assess the effect of GRP78 KD on the expression levels of N-cad in both MM and PCa we used an optimized transfection protocol suitable for suspension (MM.1S) and adherent (PC3) cell lines. A chemically modified GRP78-silencing siRNA was transfected with the TransIT-X2® dynamic delivery system that is suitable for the transfection of suspension and adherent cells [33]. The suspension nature of MM.1S cells makes these cells inherently more difficult to transfect, therefore, we doubled the siRNA concentrations (to 100 nM) and used multiple GRP78 silencing siRNAs in combination to provide a more effective GRP78 KD in these cells. While this concentration was adequately tolerated (Additional file 2), only a ~ 40% (*P* < 0.05) decrease in GRP78 protein levels was achieved, compared with ~ 70% KD (*P* < 0.001) in PC3 cells using only 50 nM of the same siRNA cocktail (Fig. 1a). Despite this difference in GRP78 KD efficiency, the MM.1S and PC3 cell lines exhibited significant decreases in N-cad protein levels although without changes in N-cad mRNA transcript levels (Fig. 1b & c). We also transfected two other MM cell lines; MM.1R and RPMI 8226, however, no significant knockdown of GRP78 was observed under optimized conditions likely due to the known challenges of transfecting non-adherent MM cells [34] (Additional file 2).

### ONCOMINE cDNA microarray analysis

The ONCOMINE microarray depository was queried for significant expression differences in the HSPA5 and CDH2 genes expressing GRP78 and N-cad, respectively in both MM and PCa, compared to normal tissue samples. All MM tumor tissue samples in these studies were accessed from BM aspirates from newly diagnosed patients. Patients in Dickens’ et al. study received autologous transplantation following induction by either a cyclophosphamide, thalidomide and dexamethasone (CTD) chemotherapy regimen for younger fit participants or cyclophosphamide, vincristine sulfate, Adriamycin, and dexamethasone (CVAD) chemotherapy regimen for older, unfit participants, while no indication of treatment was presented for the Zhan and Agnelli cohorts. The analyzed PCa tumor tissue samples ranged in origin from patients with elevated PSA (> 2.5 ng/mL) but non-confirmed cancer, prostatectomy samples, xenograft biopsies, primary organ tumor, and metastasized samples including bone, lymph node, and lung. Only one PCa trial indicated that patients had received no prior treatment. HSPA5 was overexpressed in 6/15 PCa studies (fold change ranging between 1.029–3.095, *P* < 0.05) and 2/3 MM studies (fold change of 1.095 and 1.513, *P* < 0.05). HSPA5 gene rank between 2 and 24% in PCa and was in the top 13% in the 2 MM studies with significant fold change. CDH2 was overexpressed in 1/17 PCa studies (fold change of 1.193, *P* < 0.05) and a gene rank of 14%, and 2/4 MM studies (fold change of 1.094 and 3.411, *P* < 0.05) and gene rank of 25 and 12% respectively. Results are summarized in Tables 1 and 2.

**Table 1:** ONCOMINE cDNA microarray analysis summary for MM

Multiple Myeloma					
Study	*P*-value	Fold Change	Tumor Tissue Samples	Healthy Tissue Samples	Gene Rank^a^
*HSPA5*					
Zhan	8.87E-06	1.513	74	37 (plasma)	top 13%
Dickens	1.15E-08	1.095	84	84 (leukocyte)	top 13%
*CDH2*					
Zhan	3.84E-06	3.411	74	37	top 12%
Agnelli	0.04	1.094	133	5	top 25%

ONCOMINE was searched using the following filters: Gene: HSPA5 (GRP78) or CDH2 (N-cad), Analysis Type: Cancer vs. Normal Analysis, Cancer Type: prostate cancer or multiple myeloma. ^a^Gene rank denotes the extent of significance out of all genes assayed; i.e. what percentage of genes whose upregulation has a more significant *p*-value as compared to the gene of interest

**Table 2:** ONCOMINE cDNA microarray analysis summary for PCa

Prostate Carcinoma					
Study	*P*-value	Fold Change	Tumor Tissue Samples	Healthy Tissue Samples	Gene Rank^a^
*HSPA5*					
Welsh	1.91E-07	1.958	25	9	top 2%
Singh	1.14E-04	3.095	52	50	top 4%
TCGA	1.12E-06	1.026	126	61	top 9%
	0.001	1.029	45	61	top 20%
Vanaja	0.004	1.586	27	8	top 15%
Taylor	0.013	1.169	131	29	top 17%
Grasso	0.029	1.158	59	28	top 24%
*CDH2*					
Arredouani	0.014	1.193	13	8	top 14%

ONCOMINE was searched using the following filters: Gene: HSPA5 (GRP78) or CDH2 (N-cad), Analysis Type: Cancer vs. Normal Analysis, Cancer Type: prostate cancer or multiple myeloma. ^a^Gene rank denotes the extent of significance out of all genes assayed; i.e. what percentage of genes whose upregulation has a more significant *p*-value as compared to the gene of interest

### Effect of ER stressors on GRP78 and N-cad expression levels in MM.1S cells

**Fig. 2:**
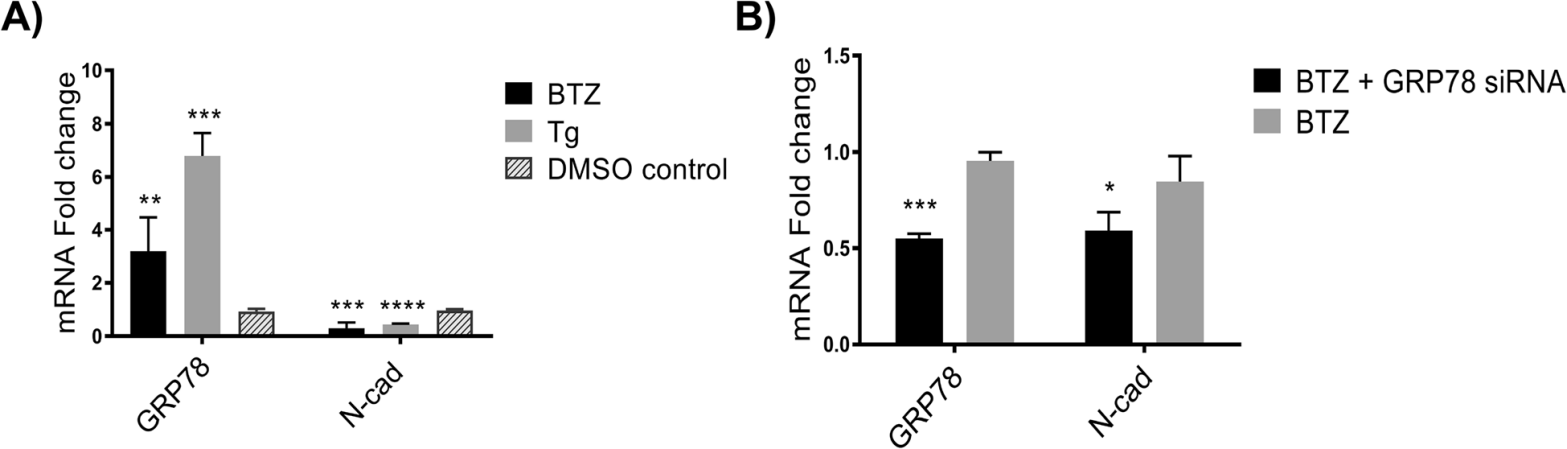
Effect of ER stress on GRP78 and N-cad mRNA levels in MM.1S cells. **a** Effect of ER stress inducers on MM.1S cells. Cells were treated with 10 nM bortezomib (BTZ) or 1 nM thapsigargin (Tg). qRT-PCR analysis was performed after 18 h of consecutive treatment. **P < 0.01 and ****P < 0.001 for GRP78 with BTZ treatment and Tg treatment vs. vehicle control (DMSO), respectively. ****P < 0.0001 for N-cad with both BTZ and Tg treatment vs. vehicle control (DMSO). **b** GRP78 silencing after BTZ treatment. MM.1S cells were treated overnight with 10 nM BTZ followed by treatment with 100 nM siRNA cocktail against GRP78. qRT-PCR analysis was performed 48 h post-transfection. ***P < 0.001 and *P* < 0.05 for GRP78 and N-cad vs. BTZ only treatment, respectively. **c** Effect of hypoxia on MM.1S cells. Cells were exposed to either 3% O_2_ for 72 h or 1% O_2_ for 24 h. Relative mRNA levels were compared to cells exposed to normoxia. ***P < 0.001 and **P < 0.01 for GRP78 (3% O_2_ and 1% O_2_). ****P < 0.0001 and **P < 0.01 for N-cad (3% O_2_ and 1% O_2_). All target mRNA levels are represented as the mean fold change ± SD of 3 separate trials

The UPR is highly upregulated in MM cells due to their secretory nature [35–37]. We sought to explore whether drug treatment or physiological ER stress-induced conditions modulate GRP78 and N-cad expression levels (Fig. 2) in the MM.1S cell line. Figure 2a shows the effects of bortezomib (BTZ), and thapsigargin (Tg) on the GRP78 and N-cad mRNA transcript levels in MM.1S cells. BTZ, a proteasome inhibitor clinically approved for the treatment of MM [36], triggered upregulation of GRP78 mRNA expression levels by ~ 3.0 fold (*P* < 0.01). Tg, an inhibitor of the Ca^2+^-ATPase that causes the accumulation of unfolded proteins and secondary ER stress [36], led to a greater (~ 7.0-fold, *P* < 0.001) increase in GRP78 mRNA transcript levels. Conversely, both ER stress inducers were found to downregulate N-cad mRNA expression levels. We next transfected BTZ treated MM.1S cells with GRP78-silencing siRNA (Fig. 2b) resulting in GRP78 mRNA KD as well as concomitant suppression of N-cad.

### GRP78 silencing has no significant effect on the expression levels of related GRPs in PC3 cells

**Fig. 3:**
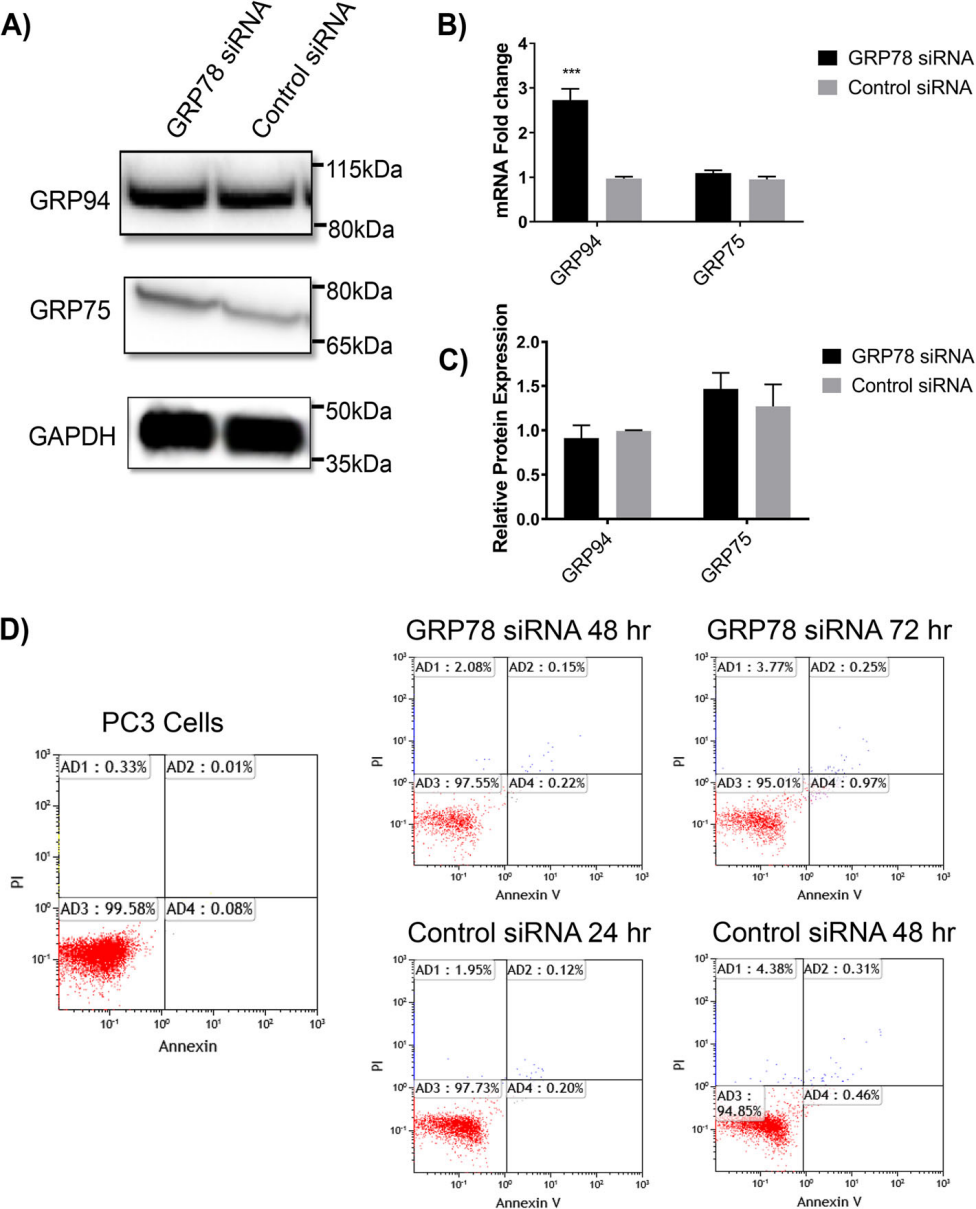
Effects of GRP78 silencing on GRP94 and GRP75 in PC3 cells. 50 nM siRNA cocktail against GRP78 or control siRNA were transfected into PC3 cells. Total mRNA and protein levels were analyzed at 48 h and 72 h, respectively. **a** Western blot of GRP protein expression after GRP78 silencing. **b** Protein expression analysis. Target protein levels were normalized against the loading control GAPDH and compared to control siRNA. Blot bands and quantitative values are presented as the mean ± SD and representative of 3 separate trials. **c** qRT-PCR analysis of relative mRNA levels of  GRPs upon GRP78 silencing. Target mRNA levels are relative to the control siRNA and represented as the mean fold change ± SD of 3 separate trials. ***P < 0.001 for GRP94. **d** Viability assay PC3 cells after siRNA transfection. 50 nM siRNA cocktail or a control siRNA were transfected into PC3 cells and analyzed by flow cytometry at 48 and 72 h. Histograms are representative of 3 independent trials at each time point

In combination with GRP78, GRP94 and GRP75 are important ER chaperones and regulators of cell homeostasis [38, 39]. Their overexpression has been linked to tumor progression, metastasis and survival in multiple cancer types [38, 39]. Because GRPs work in concert and are closely related in function, we hypothesized that GRP78 KD could potentially have an associated effect on the client chaperones GRP94 and/or GRP75. To test this hypothesis, we measured mRNA and protein levels of GRP94 and 75 upon GRP78 KD in PC3 cells. Interestingly, GRP78 KD strongly increased GRP94 mRNA levels (~ 2.7-fold, *P* < 0.001) but no changes were detected in GRP94 protein expression. GRP78 downregulation had no effect on the levels of GRP75 mRNA or protein expression (Fig. 3a,b,c).

### GRP78 silencing does not promote cytotoxicity in PC3 cells

Under severe or prolonged ER stress, the UPR has been shown to facilitate pro-apoptotic pathways ultimately leading to cell death [40]. We assessed whether the observed changes in N-cad may have been due to cellular cytotoxicity as byproduct of strong GRP78 silencing. Annexin V / PI screening by flow cytometry up to 72 h post transfection indicated no increase in cytotoxicity in relation to the control siRNA treatment (Fig. 3d).

### GRP78 silencing has significant effects on the expression of EMT related markers in PC3 cells

**Fig. 4:**
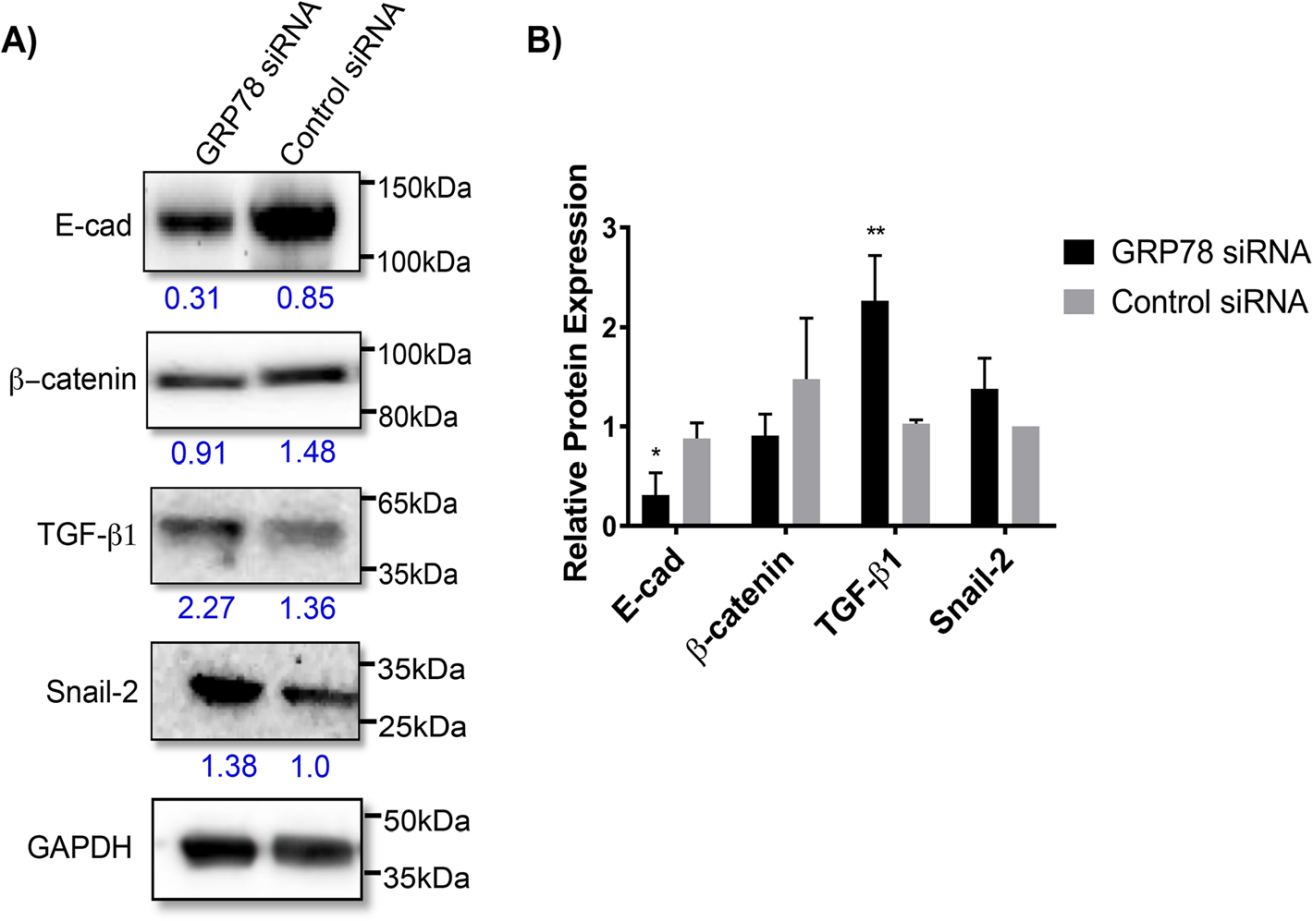
Analysis of markers related to EMT after GRP78 silencing. 50 nM siRNA cocktail against GRP78 or control siRNA were transfected into PC3 cells and total protein levels analyzed 72 h post-transfection. **a** Western blot of EMT markers and potentiators. **b** Protein expression analysis. Target protein levels were normalized against the loading control GAPDH and compared to control siRNA. Blot bands and quantitative values are presented as the mean ± SD and representative of 3 separate trials. **P < 0.01 for TGF-β1 and *P < 0.05 for E-cad

We next investigated the effects of GRP78 silencing on members of the EMT signaling pathway, in which N-cad is a major marker. The EMT of epithelial cells is partly characterized by elevated levels of N-cad, with concomitant suppression of E-cad, ultimately enabling a more migratory and invasive tumor phenotype [41]. In PC3 cells, GRP78 KD not only decreased N-cad protein expression (Fig. 1), but also that of E-cad (~ 50%, *P* < 0.05) (Fig. 4). In addition, we also investigated other EMT markers associated with PCa cells such as TGF-β and snail/slug [26]. We found a significant upregulation in TGF-β1 expression (~ 100%, *P* < 0.01), suggesting a potential role for GRP78 as an important regulator of EMT markers in metastatic PCa. While not statistically significant, changes in β-Catenin and Snail-2 were also observed and may have important biological implications (Fig. 4). Once more, due to the inherent difficulties associated with transfection of suspension cells, MM cells were not used for this part of the study.

### GRP78 silencing changes the morphology of PC3 cells and reduces their adhesiveness to OSB

**Fig. 5:**
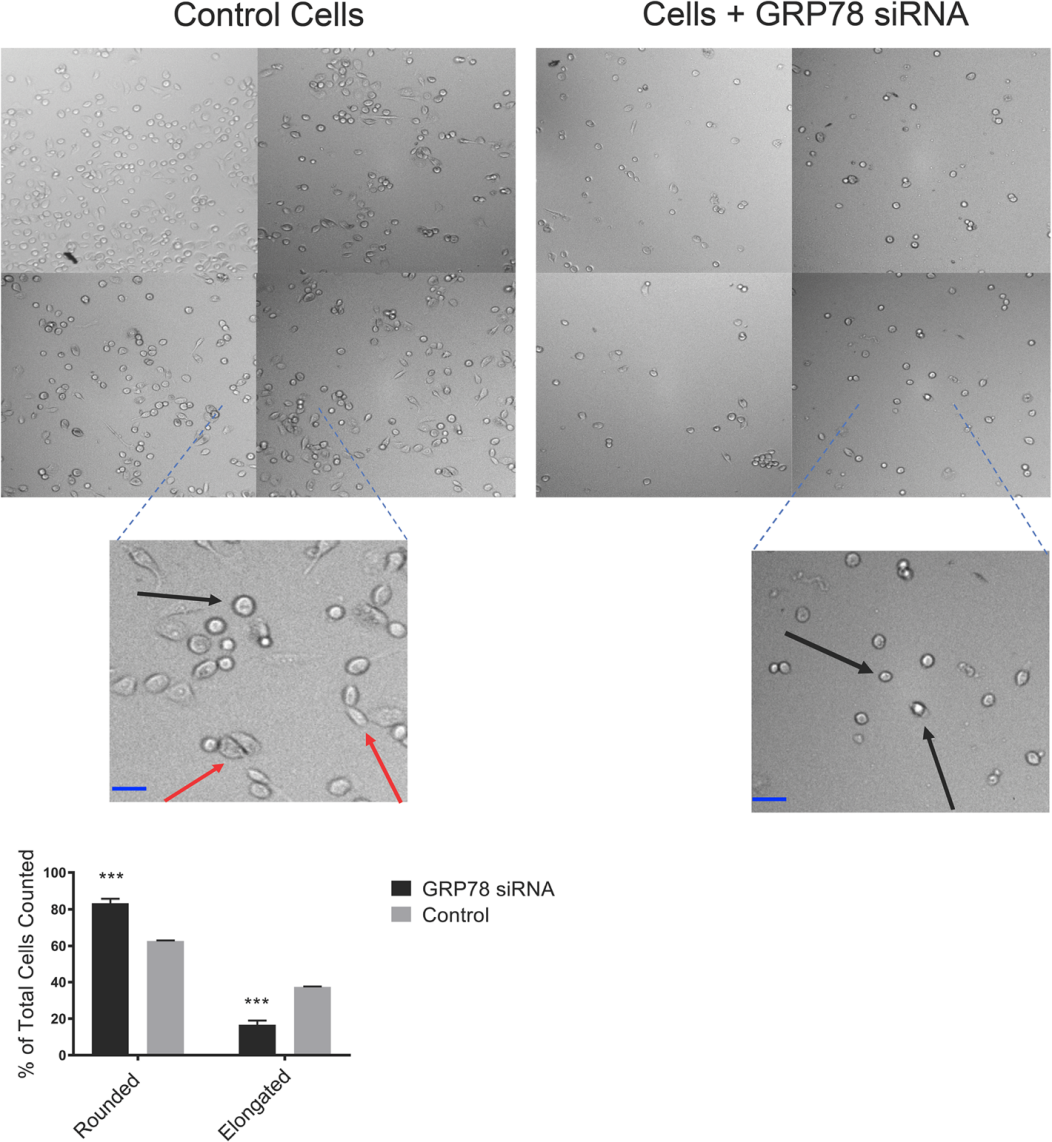
Morphological changes in PC3 cells after GRP78 KD. Cells were harvested 48 h post-transfection and re-seeded on a 24-well plate. Changes in morphology were analyzed 24 h after seeding. Bright-field microscope images (10x magnification) of cellular morphology containing 4 representative fields of view from 3 separate trials. Black arrows denote cells with rounded morphology and red arrows denote elongated cells. ***P < 0.001 compared to control (untreated) cells. Scale bar = 10 μm

**Fig. 6:**
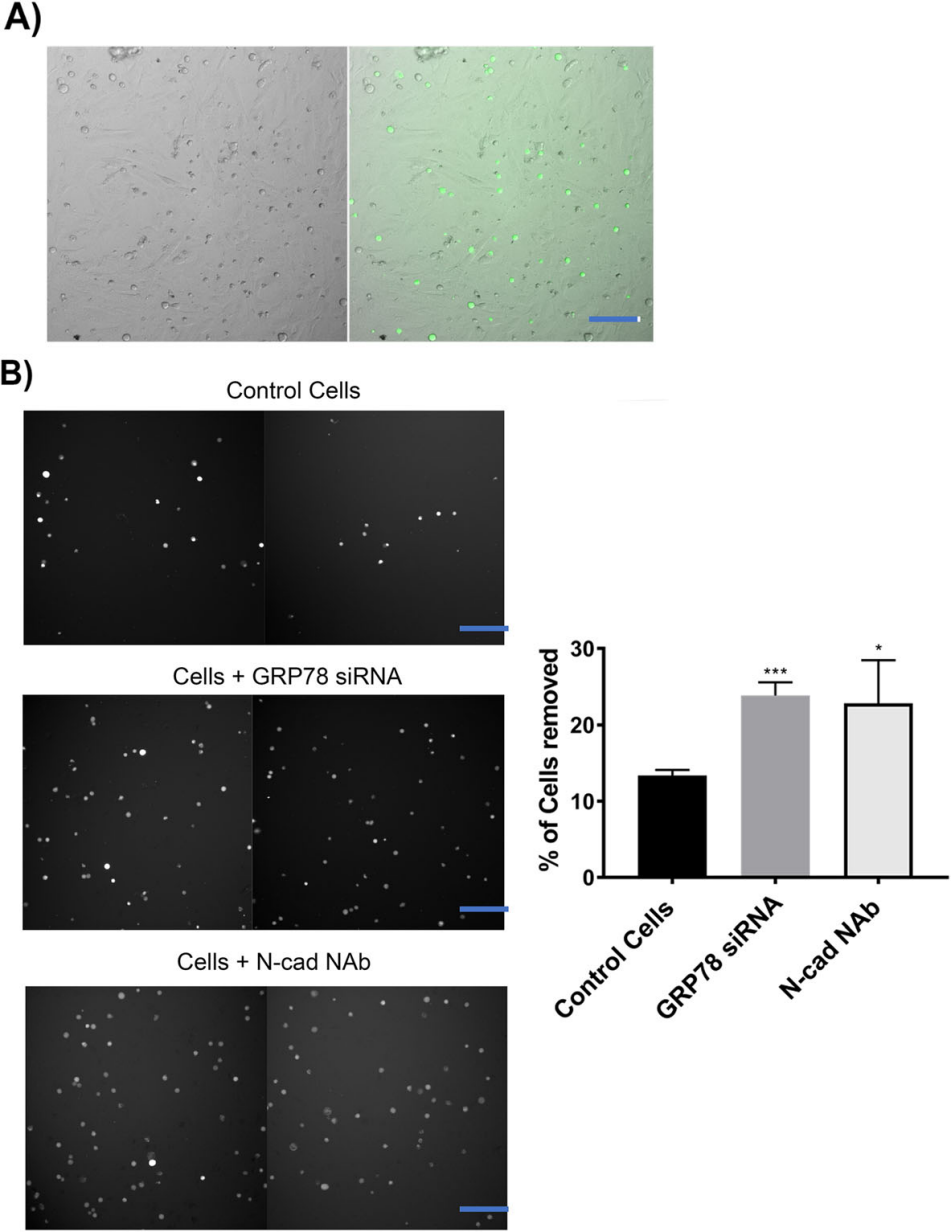
Functional changes in PC3 adherence to OSB after GRP78 KD or incubation with a N-cad NAb clone CG-4 (N-cad NAb). PC3 cells were fluorescently labeled and re-seeded into 96-well plates with confluent OSB 48 h post-transfection. Percentage of adhered cells was determined 24 h after co-culture. **a** Representative image of PC3 + OSB co-culture. Left = brightfield, right = composite. **b** Fluorescent images of labeled PC3 cells in supernatant after being removed from the co-culture and are representative of 3 independent trials. *P < 0.05 and ***P < 0.001 compared to control (untreated) cells for the N-cad NAb or the GRP78 siRNA, respectively. Scale bar = 50 μm

A co-culture assay with PC3 and hFOB 1.19 OSB cells was developed to test whether GRP78 silencing would translate into reduced adhesion to bone cells, due to the concomitant suppression of related adhesion and EMT markers. OSB were cultured to confluence in a 96-well plate. PC3 cells were initially transfected with GRP78 silencing siRNA and then cultured on top of the OSB monolayer. Parallel co-cultures were treated with the anti-N-cad mAb CG-4, known to neutralize its function [42]. Dramatic changes in morphology of the PC3 cells following GRP78 silencing were observed, indicated by a shift from a flatter, elongated shape to a rounded configuration. We also noticed a lower cell density in these wells 18 h after transfection. However, no significant changes in morphology were observed when cultures were incubated with an N-cad NAb (Fig. 5). Importantly, transfected PC3 cells were found to be less adhesive to OSB, as determined by a small but significant increase (> 10%) in the number of cells collected in the supernatant relative to the untreated control wells. Likewise, the N-cad NAb treatment reduced the co-cultured PC3 adhesion to OSB by [~ 10%] (Fig. 6b). These data support a functional role for GRP78 and N-cad in mediating cell-cell adhesive interactions in between PCa and OSB cells.

## Discussion

In this study, we describe an important correlation between GRP78, a master regulator of the UPR, and N-cad, an adhesion molecule associated with MM and PCa progression [43–45] and cell-cell adhesion implicated in drug resistance [24, 46]. N-cad expression has been shown to be directly proportional to GRP78 levels in hepatocellular carcinoma and colorectal cancer [15, 21]. Furthermore, circulating levels of N-cad have been linked to poor prognosis of MM patients [22, 23] while upregulation of this molecule was linked to PCa metastasis and castration resistance [24]. Together these findings served as basis for our hypothesis, implicating GRP78 and N-cad as important markers involved in to tumor adhesion and metastasis of PCa with bone tissue. To test this hypothesis, we investigated the effect of GRP78 KD in MM cell lines (MM.1S, MM.1R, RPMI 8226) known to reside in the BM niche and a metastatic PC3 cell line, derived from metastatic bone. Our results suggest that the pro-survival advantages conferred by GRP78 [47, 48] may also be linked to its role in modulating markers associated with cancer cell adhesion to the bone niche.

An initial survey of basal GRP78 and N-cad levels revealed comparable expressions of these proteins in MM.1S and PC3 cancer cell lines (Additional file 3, A). While GRP78 upregulation is well documented in many cancers [14, 18, 19], MM and PCa cells often display aberrant N-cad expressions [23, 24, 45, 49]. We found that GRP78 KD induced a concomitant suppression of N-cad protein levels, suggesting a regulatory relationship between these two biomarkers in MM.1S and PC3 cells (Fig. 1). Interestingly, the gene transcript levels of N-cad were not affected upon GRP78 downregulation, suggesting preferential downregulation of N-cad translation over transcriptional modulation. Importantly, only one of three MM cell lines (MM.1S) assayed showed appreciable GRP78 KD at high dose concentrations of siRNA demonstrating not only the difficulty of siRNA based gene silencing in MM, but also the dependence of MM on this important chaperone of the ER/UPR systems [37]. Alternatively, the MM.1S cells were found to be susceptible to apoptosis with the highly concentrated and long-lasting siRNA transfections (100 nM, 24-72 h) rendering further follow-up experiments unfeasible. The cancer cell lines studied (i.e., MM.1S and PC3 lines) did not manifest cell membrane bound GRP78 (data not shown) and have been shown in other reports to lack surface GRP78 under specified culture conditions [50]. Thus, our results likely indicate a role for cytosolic rather surface GRP78 on N-cad expression. However, surface expression of GRP78 has been observed in varying tumor cell lines and with ER stressors known to upregulate surface GRP78 expression [39] allowing for potential future studies aimed at investigating the effects of exogenous GRP78 blocking antibodies on N-cad activity. Likewise, additional methods [34] which fall beyond the scope of the current study may be needed to fully characterize the functional relationship in between GRP78 and N-cad among multiple MM and PCa cell lines.

We queried the Oncomine microarray repository to find any correlations in the expression profiles of HSPA5 (GRP78) and CDH2 (N-cad) in patient tissue samples. The ONCOMINE analyses revealed that both HSPA5 and CDH2 genes are overexpressed in various MM and PCa compared to normal tissue samples. In particular, HSPA5 was upregulated in 40% (6/15) and in 66% (2/3) of the clinical studies available for prostate carcinoma and MM tissues, respectively. For MM, the fold change was small likely because plasma cells and leukocytes (used as comparison samples) are secretory cells that also have high basal levels of the UPR markers [51]. CDH2 on the other hand was only significantly upregulated in one PCa study. That notwithstanding, a study describing the de novo expression of N-cad using two parameter immunofluorescence shown that this protein is expressed in high-grade human PCa, whereas no expression was found in normal prostatic tissue [52]. Likewise, circulating N-cad has also been associated with poor MM prognosis [22], supporting an underlying relationship between N-cad and GRP78 in the progression of both tumor types.

The lack of a strong transcriptional downregulation in the MM.1S cells prompted us to evaluate how the expression profiles of GRP78 and N-cad would be affected in the presence of acute drug challenge using the ER stress inducers BTZ and Tg; both of which are known to stimulate GRP78 expression and are used as current clinical treatments. We also wanted to examine whether ER stress would sensitize MM.1S cells to siRNA transfection, leading to a more robust KD effect. This strategy may be a potential clinically viable approach to facilitate the transfection of UPR dependent cancer cells. Acute drug challenge was found to induce GRP78 mRNA expression in MM.1S cells, with a concomitant decrease in N-cad mRNA levels (Fig. 2a). BTZ has been found to suppress the expression of N-cad [53] but little is known about the effects of Tg on this molecule. N-cad expression has been associated with intracellular Ca^2+^ flux, so inhibition of the ATPase-dependent Ca^2+^ flux by Tg may be correlated with its effects on N-cad transcription levels [54]. Nevertheless, when MM.1S cells were pre-treated with BTZ followed by GRP78 siRNA transfection, GRP78 KD caused an additional decrease in N-cad (Fig. 2b) gene expression. Of note, siRNA treatment did not affect genetic levels of N-cad (Fig. 1c) but the combination of ER stressors (i.e., drug treatments) and siRNA transfection did induce significant downregulation in N-cad mRNA levels, suggesting that ER stressors (such as that induced by BTZ) may sensitize MM cells to siRNA treatment.

Unlike MM, which is an exclusively BM-localized malignancy, PCa is a solid epithelial tumor that seeds and invades the bone/ BM niche. GRP78 expression has been linked to cancer progression and metastasis in part through its effects on EMT markers in PCa [55]. Induction of GRP78 has been shown to trigger EMT in colorectal cancer cells, while GRP78 KD using shRNA reversed the EMT by suppressing N-cad and upregulating E-cad expression levels, referred to as a “cadherin switch.” [21]. However, using our GRP78-silencing approach, GRP78 KD in PC3 cells resulted in significant decreases of both N-cad and E-cad (Fig. 1 & Fig. 4) protein levels. The EMT process is controlled, in part, by the transcription factor Snail-2 which acts as a strong repressor of E-cad [56, 57]. GRP78 KD induced Snail-2 expression (Fig. 4) which may account for the downregulation of E-cad.

GRP78 overexpression, its localization to the cell surface, and its association with Cripto [58, 59] have been correlated with the activation of the TGF-β pathway [60]. TGF-β is a multifunctional cytokine which regulates prostate cell growth and epithelial cell proliferation [61, 62]. However, active TGF-β exists mainly as an extracellular matrix protein which can function both as a tumor suppressor or as a key player in promoting tumorigenesis in advanced cancers [61–64]. We showed that in PC3 cells, GRP78 KD induced a strong and significant increase in TGF-β1 protein expression; consistent with our findings, induction of Snail-2 expression has been credited to TGF-β associated pathways [65]. While we observed TGF-β1 upregulation and the expected downstream effects that this molecule has on Snail-2 and E-cad, we found that GRP78 silencing decreased N-cad expression in PC3 cells. TGF-β1 upregulation is typically reported to be accompanied by N-cad upregulation [21], hence our results suggest that N-cad expression is highly dependent on GRP78 in this cell line and its regulation via GRP78 KD may supersede the effects of TGF-β1; i.e., N-cad was downregulated in spite of the fact the TGF-β1 expression was significantly increased upon GRP78 KD. This may indicate a unique mechanism in which GRP78 KD can directly modulate the expression of certain adhesion and EMT molecules. We also showed that downregulation of GRP78 did not change the protein expression levels of other chaperone GRPs nor cause cytotoxicity in PCa. These results confirm that the observed effects on the EMT markers were GRP78-dependent and not the result of global changes in closely related chaperones that also maintain ER homeostasis or the result of apoptosis.

TGF-β has also been implicated in EMT signaling through alternative non-smad pathways. For example, Ras homolog gene family member A (RhoA)-dependent signaling is activated by TGF-β and induces mesenchymal characteristics in epithelial cells [66]. Signaling by RhoA and its effector proteins Rho kinase-ROCKI and ROCKII promote amoeboid movement of tumor cells and the adoption of a more rounded shape [67]. Consistent with these morphological changes, we observed that following GRP78 KD, PC3 cells underwent a shift from a flatter, elongated shape to a more rounded configuration (Fig. 5) Importantly, incubation with a N-cad NAb did not significantly change the morphology of the cells (Additional file 3, B) implying that the observed changes were directly related to the diminished GRP78 levels rather than a function of N-cad activity.

We suspected that N-cad downregulation upon GRP78 KD could lead to reduced adhesion of PCa to the bone microenvironment. We confirmed this hypothesis by co-culturing PC3 cells transfected with GRP78 silencing siRNAs with a monolayer of OSB cells. The cells were found to be less adherent to OSB, relative to untreated control cells, supporting the hypothesis that GRP78 KD and suppression of N-cad could significantly inhibit PCa cell-based adhesion to bone. In a comparable manner N-cad NAb treatment also diminished PCa adhesion to OSB (Fig. 6). That notwithstanding, other adhesion molecules have been reported to play a role in PCa-bone interaction [68], potentially accounting for the only moderate decrease in PC3 adhesion to OSB in our assays.

## Conclusion

We have established a novel correlation between GRP78 and N-cad in MM and PCa cells and present GRP78 as an ancillary regulator of markers associated with the EMT pathway and its implications in the adhesion properties of PCa to the bone/BM niche. Aside from the extensively described roles in tumor progression, our data suggest that downregulation of GRP78 may represent a suitable therapeutic intervention strategy for modulating tumor-microenvironment adhesive interactions leading to tumor progression and drug resistance. In summary, our results warrant additional investigations to further unravel the molecular interactions by which GRP78 asserts adhesion regulation, given its primary localization and role as a master regulator of the UPR in the ER.

## Additional files

Additional file 1: List of cDNA microarray sources used in ONCOMINE analysis. Study references from which the ONCOMINE data was sourced. (PDF 66 kb)

Additional file 2: Viability assay of MM.1S cells after siRNA transfection a) GRP78 silencing in MM.1R and RPMI 8226 MM cell lines. 100 nM siRNA cocktail against GRP78 or a control siRNA were transfected into Each cell line. Total mRNA levels were analyzed at 48 h. qRT-PCR analysis of relative mRNA levels for GRP78 and N-cad upon GRP78 silencing. Target mRNA levels are relative to the control siRNA and represented as the mean fold change ± SD of 3 separate trials. b) Viability assay of MM.1S cells after siRNA transfection. 100 nM siRNA cocktail or a control siRNA were transfected into MM.1S cells and analyzed by flow cytometry over 72 h. Histograms are representative of 3 independent trials at each time point. (TIF 58549 kb)

Additional file 3: Basal expression levels of GRP78 and N-cad in MM.1S and PC3 cell lines. a) Basal expression levels of GRP78 and N-cad in MM.1S and PC3 cell lines. Western blot shows comparable expression levels GRP78 compared to N-cad in MM.1S and PC3 cell lines. Expression levels were normalized to the loading control, GAPDH, and expressed as relative units. Blot bands are representative of 3 separate trials. Western blot analysis for MM.1S and PC3 cells were performed independently. b) Morphological changes in PC3 cells after incubation with the N-cad NAb, clone CG-4. Bright-field microscope images (10x magnification) of cellular morphology containing 4 representative fields of view from 3 separate trials. Scale bar = 10 μm. (TIF 55325 kb)

## Abbreviations

ATCC: American Type Culture Collection
BM: Bone Marrow
BTZ: Bortezomib
E-cad: E-cadherin
ER: Endoplasmic Reticulum
GRP78: 78 kDa Gucose-Regulated Protein
MM: Multiple Myeloma
N-cad: N-cadherin
OSB: Osteoblasts
PCa: Prostate Cancer
qRT-PCR: quantitative Reverse Transcription and Polymerase Chain Reaction
SD: Standard Deviation
siRNA: Small interfering RNA
Tg: Thapsigargin
UPR: Unfolded Protein Response
