# Supplementary material for: GRP78 modulates cell adhesion markers in prostate Cancer and multiple myeloma cell lines
Source: BMC Cancer. 2018 Dec 18;18:1263. doi: 10.1186/s12885-018-5178-8 (PMC6299583; doi:10.1186/s12885-018-5178-8)
Supplement: Supplementary file 1 — List of cDNA microarray sources used in ONCOMINE analysis. Study references from which the ONCOMINE data was sourced. (PDF 66 kb) [file 12885_2018_5178_MOESM1_ESM.pdf]

List of cDNA microarray sources used in ONCOMINE analysis
(1-10)

1. Agnelli L, M. L., Fabris S, Lionetti M, Andronache A, Kwee I, Todoerti K, Verdelli D, Battaglia C, Bertoni F, Deliliers GL, Neri A. (2009) A SNP microarray and FISH-based procedure to detect allelic imbalances in multiple myeloma: an integrated genomics approach reveals a wide gene dosage effect. *Genes Chromosomes Cacner* **48**, 603-614
2. Arredouani MS, L. B., Bhasin M, Elijanne M, Yue W, Mosquera JM, Bubley GJ, Li V, Rubin MA, Libermann TA, Sanda MG. (2009) Identification of the transcription factor single-minded homologue 2 as a potential biomarker and immunotherapy target in prostate cancer. *Clin Cancer Res* **15**
3. Dickens NJ, W. B., Leone PE, Johnson DC, Brito JL, Zeisig A, Jenner MW, Boyd KD, Gonzalez D, Gregory WM, Ross FM, Davies FE, Morgan GJ. (2010) Homozygous deletion mapping in myeloma samples identifies genes and an expression signature relevant to pathogenesis and outcome. *Clin Cancer Res* **16**, 1856-1864
4. Grasso CS, W. Y., Robinson DR, Cao X, Dhanasekaran SM, Khan AP, Quist MJ, Jing X, Lonigro RJ, Brenner JC, Asangani IA, Ateeq B, Chun SY, Siddiqui J, Sam L, Anstett M, Mehra R, Prensner JR, Palanisamy N, Ryslik GA, Vandin F, Raphael BJ, Kunju LP, Rhodes DR, Pienta KJ, Chinnaiyan AM, Tomlins SA. (2012) The mutational landscape of lethal castration-resistant prostate cancer. *Nature* **487**, 239-243
5. Singh D, F. P., Ross K, Jackson DG, Manola J, Ladd C, Tamayo P, Renshaw AA, D'Amico AV, Richie JP, Lander ES, Loda M, Kantoff PW, Golub TR, Sellers WR. (2002) Gene expression correlates of clinical prostate cancer behavior. *Cancer Cell* **1**, 203-209
6. Taylor BS, S. N., Hieronymus H, Gopalan A, Xiao Y, Carver BS, Arora VK, Kaushik P, Cerami E, Reva B, Antipin Y, Mitsiades N, Landers T, Dolgalev I, Major JE, Wilson M, Socci ND, Lash AE, Heguy A, Eastham JA, Scher HI, Reuter VE, Scardino PT, Sander C, Sawyers CL, Gerald WL. (2010) Integrative genomic profiling of human prostate cancer. *Cancer Cell* **18**, 11-22
7. Vanaja DK, C. J., Iturria SJ, Young CY. (2003) Transcriptional silencing of zinc finger protein 185 identified by expression profiling is associated with prostate cancer progression. *Cancer Res* **63**, 3877-3882
8. Welsh JB, S. L., Su AI, Kern SG, Wang-Rodriguez J, Moskaluk CA, Frierson HF Jr, Hampton GM. (2001) Analysis of gene expression identifies candidate markers and pharmacological targets in prostate cancer. *Cancer Res* **61**, 5974-5978
9. Zhan F, H. J., Kordsmeier B, Bumm K, Zheng M, Tian E, Sanderson R, Yang Y, Wilson C, Zangari M, Anaissie E, Morris C, Muwalla F, van Rhee F, Fassas A, Crowley J, Tricot G, Barlogie B, Shaughnessy J Jr. (2002) Global gene expression profiling of multiple myeloma, monoclonal gammopathy of undetermined significance, and normal bone marrow plasma cells. *Blood* **99**, 1745-1757
10. The Cancer Genome Atlas- Prostate Adenocarcinoma DNA Copy Number Data
