# Supplementary figures and images for: GRP78 modulates cell adhesion markers in prostate Cancer and multiple myeloma cell lines
Source: BMC Cancer. 2018 Dec 18;18:1263. doi: 10.1186/s12885-018-5178-8 (PMC6299583; doi:10.1186/s12885-018-5178-8)

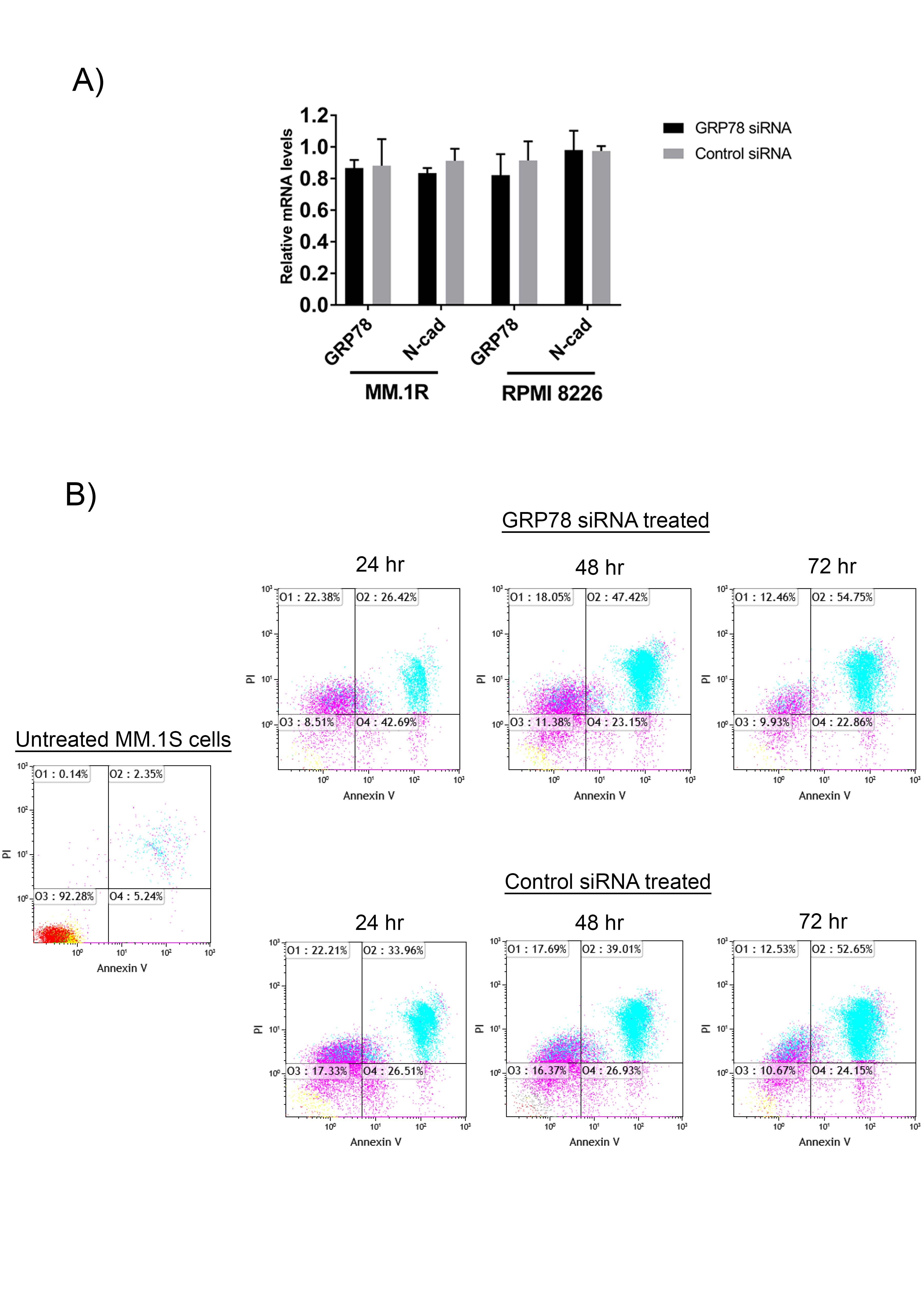

Supplement: Supplementary file 2 — Viability assay of MM.1S cells after siRNA transfection a) GRP78 silencing in MM.1R and RPMI 8226 MM cell lines. 100 nM siRNA cocktail against GRP78 or a control siRNA were transfected into Each cell line. Total mRNA levels were analyzed at 48 h. qRT-PCR analysis of relative mRNA levels for GRP78 and N-cad upon GRP78 silencing. Target mRNA levels are relative to the control siRNA and represented as the mean fold change ± SD of 3 separate trials. b) Viability assay of MM.1S cells after siRNA transfection. 100 nM siRNA cocktail or a control siRNA were transfected into MM.1S cells and analyzed by flow cytometry over 72 h. Histograms are representative of 3 independent trials at each time point. (TIF 58549 kb) [file 12885_2018_5178_MOESM2_ESM.tif]

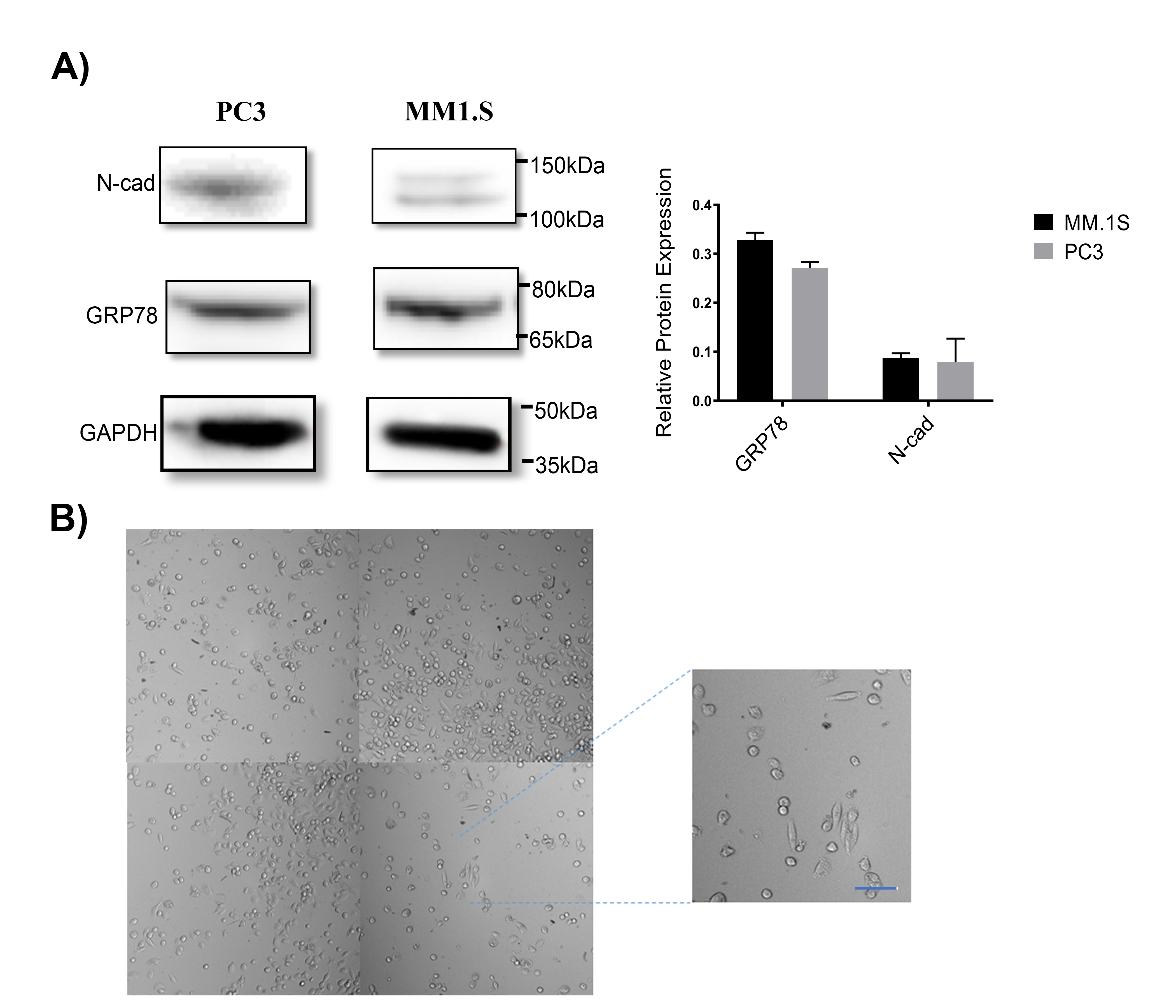

Supplement: Supplementary file 3 — Basal expression levels of GRP78 and N-cad in MM.1S and PC3 cell lines. a) Basal expression levels of GRP78 and N-cad in MM.1S and PC3 cell lines. Western blot shows comparable expression levels GRP78 compared to N-cad in MM.1S and PC3 cell lines. Expression levels were normalized to the loading control, GAPDH, and expressed as relative units. Blot bands are representative of 3 separate trials. Western blot analysis for MM.1S and PC3 cells were performed independently. b) Morphological changes in PC3 cells after incubation with the N-cad NAb, clone CG-4. Bright-field microscope images (10x magnification) of cellular morphology containing 4 representative fields of view from 3 separate trials. Scale bar = 10 μm. (TIF 55325 kb) [file 12885_2018_5178_MOESM3_ESM.tif]
